# Cost-Effectiveness Analysis of Medications in the Field of Sexual Medicine Offered by the Brazilian Universal Healthcare System at a Tertiary Hospital

**DOI:** 10.7759/cureus.93772

**Published:** 2025-10-03

**Authors:** André Luiz de Godoi, Fernando Nestor Facio, Luís Cesar Fava Spessoto, Pedro Arrruda, Gisela Cipullo Moreira, Moacir Godoy, Jose Maria Pereira de Godoy

**Affiliations:** 1 Faculty of Health Sciences, Faculty of Medicine of São José do Rio Preto (FAMERP), São José do Rio Preto, BRA; 2 Urology, Faculty of Medicine of São José do Rio Preto (FAMERP), São José do Rio Preto, BRA; 3 Clinical Pharmacy, Fundação Faculdade Regional de Medicina de São José do Rio Preto (FUNFARME), São José do Rio Preto, BRA; 4 Cardiology, Faculty of Medicine of São José do Rio Preto (FAMERP), São José do Rio Preto, BRA; 5 Cardiology and Cardiovascular Surgery, Faculty of Medicine of São José do Rio Preto (FAMERP), Sao Jose do Rio Preto, BRA

**Keywords:** access to medications, judicialization of health, pharmaceutical assistance, sexual health, universal healthcare system

## Abstract

Medicine is an indispensable component of healthcare systems. However, ensuring its availability, accessibility, rational use, cost-effectiveness, and sustainability poses challenges for most countries throughout the world. The present study involved a retrospective analysis of 7,929 medical appointments recorded in electronic medical records from 2016 to 2020 at a general specialty outpatient clinic of a tertiary-level teaching and research hospital in Brazil. A total of 1,377 prescriptions were issued for medications in the field of sexual medicine not available through the universal healthcare system, with a focus on erectile dysfunction (ED), offered to 1,015 patients. The results revealed sustainable ways of treating patients in specialized care with more complex drug therapy, even if such drugs are not on the National List of Essential Medications. This study demonstrated the importance of management with a focus on communication and synergistic interaction among healthcare providers. Medications for ED within the scope of the universal healthcare system, even if not available through pharmaceutical assistance programs, had a low rate of access through the courts (0.8%) compared to other specialties, but were offered in a cost-effective manner.

## Introduction

Medications constitute an indispensable component of healthcare systems. However, ensuring their availability, accessibility, rational use, cost-effectiveness, and sustainability poses challenges for most countries throughout the world, especially given the progressive increase in demand [1].

Access to medicines and their rational use in Brazil is ensured by the universal healthcare system, which is considered one of the largest, with important advances over the years to promote access, such as the National Medicines Policy and National pharmaceutical assistance Policy, which were approved in 1998 and 2004, respectively, after the publication of Decree 7,508, and the creation of the National Commission for the Incorporation of Technologies into the universal healthcare system in 2011. The guidelines established in the National Medicines Policy include the definition of a list for the public supply of medicines - the National List of Essential Medicines - and the orientation that prescriptions comply with the therapeutic guidelines defined in the clinical protocol for the disease or health problem in question [2-4]. According to the World Health Organization, the appropriate use of essential medicines is one of the most cost-effective components of modern health care [5].

The National Policy for Comprehensive Men’s Health Care was established in 2009 and covers specific aspects of sexual medicine, advocating the treatment of erectile dysfunction (ED), which is considered the most prevalent sexual disorder. Even 20 years after the establishment of this policy, however, we have not yet seen the incorporation of specific medications for the treatment of ED on the National List of Essential Medicines [6].

The World Health Organization defines sexual health as a physical, emotional, mental, and social state of well-being in relation to sexuality. It is not merely the absence of disease, dysfunction, or debility [7]. An estimated 50% of men over 40 years of age have ED, which can reach 90% by the age of 70. The incidence could reach 322 million cases in 2025 [8]. According to the Brazilian Society of Urology, this problem becomes more common as men age. Moreover, recent studies indicate that it may be an early warning sign of a more serious problem, such as cardiovascular disease, diabetes, stroke, or circulatory problems [9,10].

The demand for medium- and high-complexity services related to men’s health has increased exponentially. This increase may be attributed to the hospital-centric culture as well as improvements in the provision of services through regulatory policies [11]. The increase in the number of medical appointments in specialized care has been accompanied by a gradual increase in the number of medical prescriptions offered to patients, offering health technologies with greater technological density. However, the vast majority of these technologies are not yet available through the universal healthcare system, such as medications indicated for the treatment of ED. Consequently, access to these technologies may occur through the judicial route. Given this scenario, it is important to develop strategic, cost-effective actions in the secondary care level of the universal healthcare system with a high number of appointments to offer medical prescriptions to patients, providing access to and the rational use of medications with sustainability for the healthcare system [12].

Resolution SS-54 of 2012 was published in the state of São Paulo, which established the Pharmacology Commission of the São Paulo State Secretary of Health, whose main objective is to assess administrative requests for medications not available through the universal healthcare system sent by patients [13]. However, Resolution SS-83 of August 17, 2015, was published in the state of São Paulo, which establishes penalties for state health services that issue prescriptions for medications not available through the universal healthcare system [14].

It is important to stress that, although ED medications are not available through the universal healthcare system, when prescribed, access must be ensured in line with basic principles, especially patient safety [15]. Treatment for ED can be divided into first line (PDE5i inhibitors (oral treatment), hormone therapy, and/or psychotherapy), second line (intracavernous self-injection), and third line (penile implant) [16].

The availability of medicines through the universal healthcare system, according to the National List of Essential Medicines, occurs through the basic, strategic, and specialized components of pharmaceutical assistance, each of which has different characteristics (form of organization, funding, and list of medicines) as well as different criteria for access to and the availability of drugs [6].

It is important to present prescribers working in the universal healthcare system with the therapeutic path that should be followed in order to provide patients with access to the proposed treatment. When prescribing medication, prescribers are subject to several influences, such as conceptions about the health-disease process, the quality of technical training, sociocultural and economic aspects of the population served, the availability of medicines in the service at which he/she work, and harassment from the pharmaceutical industry [17].

The aim of this article was to analyze the cost-effectiveness of medications in the field of sexual medicine offered by the universal healthcare system at a tertiary hospital considering 1) medical prescriptions offered in outpatient care and upon discharge from hospital, 2) prescriptions for medications not available through the universal healthcare system originating from the urology/men’s health service, and 3) the judicialization of medications available and not available through the universal healthcare system.

## Materials and methods

A quantitative, retrospective, cross-sectional, cost-effectiveness study was conducted involving patients who attended medical appointments at the General and Specialty Outpatient Clinic of a tertiary hospital in Brazil from January 2016 to December 2020. The patients were treated exclusively under the urology/sexual medicine specialty and received prescriptions containing medications indicated for the treatment of sexual dysfunction, specifically ED.

The study was developed at the General and Specialty Outpatient Clinic, which is an integral part of the Regional Medical School Foundation Complex of the São José do Rio Preto Base Hospital in the state of São Paulo, Brazil. The outpatient clinic has approximately 30,000 appointments/month, 1,600 of which are related to general urology and specifically for patients treated at the men's health/sexual medicine outpatient clinic, with approximately 1,596 appointments recorded annually, 133 monthly, and 33 weekly.

Data were obtained through a rigorous analysis of all medical prescriptions originating from in-person outpatient care for patients of the universal healthcare system, whose data were included in electronic records during medical appointments. These data were available in the computerized program/system denominated MVPEP - Soul MV - (SMA-PEP version 2020.011.07) in its most recent update.

A report was first generated by the Soul MV system from January 2016 to December 2020 to visualize all the appointments at the outpatient clinic characterized as the urology/sexual medicine/men’s health specialty, with a focus on the underlying condition (ED). Only patients who attended outpatient appointments on Wednesday mornings, which is the specific day and time for the sexual medicine/men’s health subspecialty, were selected. After generating the report on the computer screen, the 681 pages of the PDF file were copied and exported to a Microsoft Office Excel 2007 spreadsheet.

The reports were stratified for each year of interest (2016, 2017, 2018, 2019, and 2020), considering the male population (excluding duplicate patients) and those who underwent medical follow-up in the same year. A number was assigned to each medication described in the prescription.

The following information was entered onto the Microsoft Office Excel spreadsheet: patient’s name, medical record number, year of appointment, age range, description of the medications prescribed per patient, and the number of prescriptions containing specific medications for ED, as proposed in the objective of this study (testosterone cypionate, testosterone undecanoate, testosterone undecylate, sildenafil, tadalafil, and alprostadil).

The data in the Soul MV report also included all services provided in the post-appointment Document Screening Sector for Access to Medicines in the period from 2016 to 2020. To analyze prescriptions for medications for the treatment of ED not available through the universal healthcare system, the 2022 National List of Essential Medicines was adopted as a reference.

The dispensing of medications available and those not available through the universal healthcare system that were subject to legal proceedings between 2016 and 2020 was verified by analyzing the judicialization spreadsheet received monthly by e-mail from the financial superintendence of the institution, which receives it from the Strategic Demands Coordination Group of the universal healthcare system. The first, second, third, and sixth months of 2016, the eighth month of 2018, and the eighth month of 2019 were not considered in this spreadsheet analysis due to a lack of information. However, the Integrated Citizen Information System of the São Paulo State Secretary of Health was electronically requested to provide the list of all medications dispensed to patients through legal action with prescriptions originating from the institution in order to verify the data.

The 15th Regional Health Department in the municipality of São José do Rio Preto was also asked to provide receipts issued by the management system S-Codes software, which is a computerized system for handling legal action cases for the judicialized medications whose dispensation was consolidated, as observed in the names of the authors informed in the judicialization spreadsheet. For the purposes of this study, only the judicialization of demands against the state was considered.

Qualitative variables and discrete quantitative variables were presented as quantities and percentages. Inferential analysis was performed using contingency tables and Fisher's exact test. An alpha error of 5% was accepted, with P values less than or equal to 0.05 being considered significant. The results were illustrated using column charts and line charts. The software used was Stats Direct version 3.3.5 (03/22/2021) - Stats Direct Limited - Wirral, UK.

## Results

According to the report extracted from the Soul MV software, 7,929 appointments were recorded. Filters were applied, excluding 1,070 appointments related to female patients who had appointments in another subspecialty on the same day and 2,610 duplicate appointments. Among the 4,249 appointments analyzed, 1,015 were specific to patients diagnosed with ED. A total of 1,377 prescriptions containing one of the medications for the treatment of ED (testosterone cypionate, testosterone undecanoate, sildenafil, tadalafil, and alprostadil) and one penile implant were offered to the 1,015 patients. A total of 1,313 prescriptions were issued for treatment with first-line medications, as some patients took 2/3 medications (e.g., testosterone undecylate; tadalafil and sildenafil); 72 patients received the second line of treatment, and 57 patients (not recorded in this study) received the third line of treatment (penile implant) (Table 1).

**Table 1:** Prescriptions offered to patients for medications X line of treatment.

First Line of Treatment		
	Prescription	%
Sildenafil 100 mg	107	7.42
Sildenafil 20 mg	3	0.21
Sildenafil 25 mg	2	0.14
Sildenafil 50 mg	224	15.53
Tadalafil 10 mg	39	2.70
Tadalafil 20 mg	247	17.13
Tadalafil 40 mg	1	0.07
Tadalafil 5 mg	655	45.42
Tadalafil 50 mg	4	0.28
Tadalafil 8 mg	2	0.14
Testosterone cypionate 200 mg/2 ml	15	1.04
Testosterone propionate 30 mg + testosterone phenpropionate 60 mg + testosterone isocaproate 60 mg+ testosterone decanoate 100 mg 250 mg/ml	1	0.07
Testosterone undecanoate 250 mg/ml	13	0.90
Total	1313	91
Second line of treatment		
Alprostadil (10 mcg or 20 mcg)	72	4.99
Total	72	5
Third line of treatment		
Penile implant	57	3.95
Total	57	4

The quantity of medications distributed over time (2016 to 2020) was compared relatively in two age groups: less than 60 years and 60 years or older (Table 2).

**Table 2:** Age groups of patients who received prescriptions for the treatment of ED in the period from 2016 to 2020

Age	No of Patients	%
> 90	2	0.15
14 to 19	1	0.07
20 to 29	4	0.29
30 to 39	25	1.82
40 to 49	103	7.48
50 to 59	312	22.66
60 to 69	555	40.31
70 to 79	335	24.33
80 to 89	40	2.90
Total	1377	100

Comparison - 2016 versus 2020

**Figure 1:**
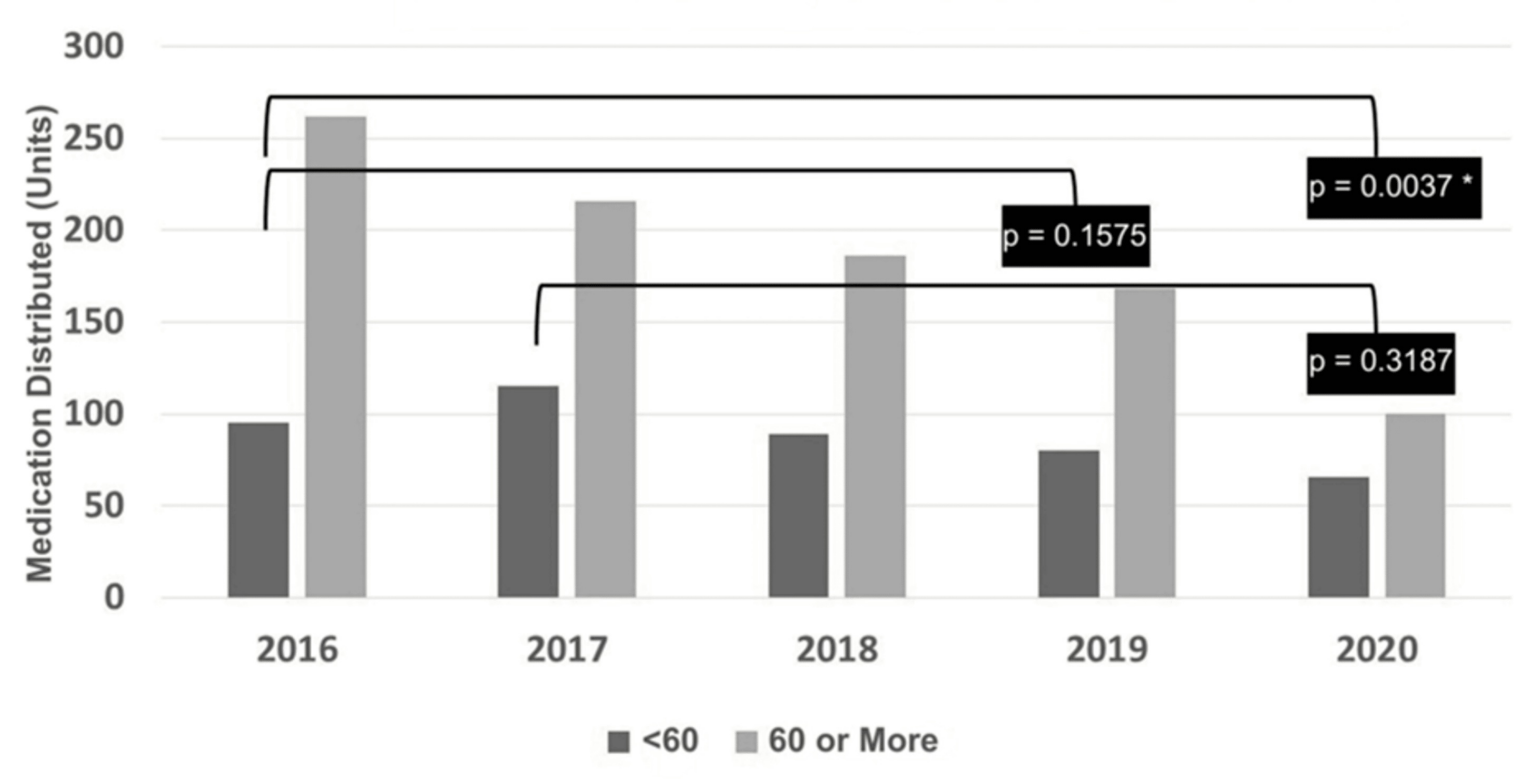
Evolution of quantity of medications distributed per age group (<60 years versus ≥60 years) over the years (Fisher’s exact test). *Statistically significant difference (p<0.05)*Statistically significant difference (p<0.05).

A significantly greater number of individuals 60 years of age or older received medications in 2016 compared to 2020 (p = 0.0037, Fisher’s exact test) (Figure 1).

Other comparisons

A statistically significant difference was found in the quantity of medicines received according to age group in 2016 versus 2020 (p = 0.0037, Fisher’s exact test). No statistically significant difference was found in the quantity of medications received according to age group in 2016 versus 2019 (p = 0.1575, Fisher’s exact test). No statistically significant difference was found in the quantity of medicines received according to age group in 2017 versus 2020 (p = 0.3187, Fisher’s exact test) (Figure 1).

Specifically in the Document Screening Sector for Access to Medicines, 244 follow-up appointments occurred to make corrections and adjustments to administrative processes received for the promotion of access to the proposed treatment. A total of 173 administrative processes were checked, corresponding to 71% of all processes and consisting of documents essential for access to medications, such as prescriptions, specific medical exams necessary to prove the disease according to the medication requested, the scheduling of follow-up appointments to request medicines, etc.).

Analyzing the judicialization of medications for the treatment of ED, the 1,377 prescriptions offered for treatment to 1,015 patients were not available in the universal healthcare system. Only 0.8% of these prescriptions for ED medications were upheld through legal action (Table 3).

**Table 3:** Annual discounts imposed on the institution referring to health technologies acquired through legal action.

Yes	Judicialization Value	
	Other specialties	Urology (sexual dysfunction)
2016	R$ 225,424.72	R$ 0.00
2017	R$ 555,278.96	R$ 248.61
2018	R$ 233,974.51	R$ 706.44
2019	R$ 232,882.07	R$ 27.15
2020	R$ 187,555.13	R$ 180.00
Total	R$ 1,435,115.39	R$ 1,162.20
Total judicialized items	R$ 1,436,277.59	

**Figure 2:**
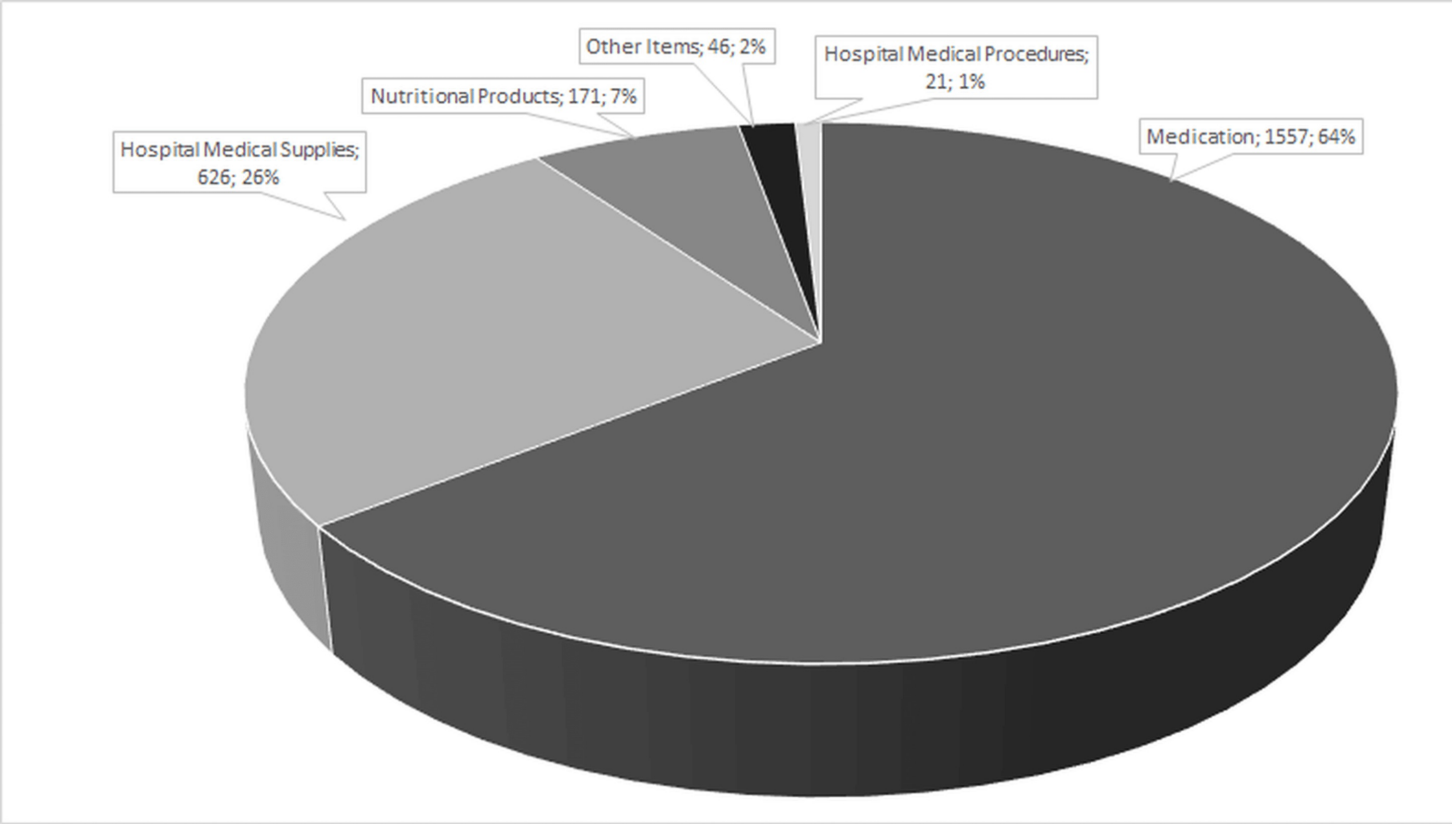
Frequency of judicialized items with prescription. Source: Health Technology Evaluation Center-Regional Medical School Foundation

Three (n = 3, 100%) patients acquired medications for the treatment of ED through legal means: one (n = 1, 33,3%) for tadalafil 5 mg and two (n = 2, 66,7%) for testosterone. Each patient received the medication more than once. It is clear that medications are the items most subject to legal proceedings. For the purposes of this study, only pharmacotherapy was considered (Figure 2).

The Health Technology Evaluation Center forwarded 249 administrative requests containing medications for ED originating from medical care at the general urology outpatient clinic to the Pharmacology Commission of the São Paulo State Secretary of Health for technical evaluation in the period studied. The total number of administrative processes, known as other demands, arising from prescriptions of medicines not available through the universal healthcare system in the period from 2016 to 2020 sent to the Pharmacology Commission for evaluation resulted in (n = 212, 85%) of the processes being granted, (n = 35, 14%) of the processes being denied, and (n = 2, 1%) being returned. No incidents of approval by the Pharmacology Commission of the São Paulo State Secretary of Health occurred with regard to prescriptions for short-acting hormone replacement drugs.

## Discussion

Analyzing the number of prescriptions, 1,377 were offered to 1,015 users in outpatient care from 2016 to 2020 for the treatment of ED. This was considered relevant, as these are technologies not available in any of the pharmaceutical assistance programs of the universal healthcare system. Moreover, it is noteworthy that these prescriptions were made in specialized care in a cost-effective manner through monitoring, simultaneously providing the patient with rational access to the medication and the sustainability of the healthcare system.

Patients 60 years of age or older received the largest number of prescriptions for ED medications, totaling 628 prescriptions and corresponding to 62% of the total. This result is in agreement with the data published in the most epidemiologically important study on the subject due to its rigorous methodology - the Massachusetts Male Aging Study conducted between 1987 and 1989 [18]. In contrast, this does not match what is recommended by the National Policy for Comprehensive Men’s Health Care, which states the greatest number of appointments should occur in the population aged between 25 and 59 years, preferably in primary care [19].

The present study shows that the male population seeks medical care in the most severe phase of the disease, which ends up increasing costs for the healthcare system. This is demonstrated by the number of appointments offered to patients 60 years of age or older and, consequently, the number of prescriptions offered, totaling 555 prescriptions (40.31% of the total) at a specialized service considered a reference for medium- and high-complexity treatment. This scenario points to a contradiction between the National Policy for Comprehensive Men’s Health Care and the National Primary Care Policy, which should be aligned to enable better outcomes in the healthcare process [19]. Primary care should be used as the gateway to the universal healthcare system, offering greater resolution and humanized strategies in line with the principles of the universal healthcare system and strengthening actions and services in healthcare networks [20].

It is important to point out that the prescriptions analyzed in this study were largely written by resident physicians under the supervision of supervising physicians. Thus, offering 1,377 prescriptions for medications not available in the universal healthcare system for the treatment of ED required effective monitoring and assistance in the writing of the prescriptions to ensure compliance with medical ethics and the guidelines of the universal healthcare system. To achieve this, it is important to provide prescribers with information technologies. In the present study, the creation of a systems integrator was observed, providing medical prescriptions in accordance with the National List of Essential Medicines and current legislation.

Healthcare providers often have little time for medical appointments. Physicians need to provide patients with a series of documents to promote access to medications. As this is an outpatient service, prescriptions and other documents offered to the same patient may contain medications available in several pharmaceutical assistance programs simultaneously (People’s Pharmacy of Brazil, basic, strategic, and specialized components of pharmaceutical assistance, oncology, and dispensing pharmacies), each of which has its own specificities [21,22].

The synergistic action between physicians and pharmacists was considered important. Pharmacists can align the process of preparing prescriptions and other documents with the policies of the universal healthcare system, while also providing assistance to prescribers and, consequently, promoting patient engagement and access to the proposed treatment in a rational manner. Several studies have shown that including pharmacists on multidisciplinary teams results in more cost-effective outcomes, but most of these studies were aimed at studying a specific population, disease, or situation [23].

This study recognized the importance of offering post-appointment care, as observed in the Document Screening Sector for Access to Medicines. Healthcare providers exhibited technical operational skills under the supervision of the pharmacist, providing access to the proposed treatment in a cost-effective manner. A humanized approach was found in this sector, with the checking of documents originating from medical appointments and the provision of information on the paths (therapeutic itinerary) to be followed to obtain access to medicines that are often impeded due to a lack of information and knowledge [24].

For state-run healthcare institutions, offering prescriptions for more complex medications not available through the universal healthcare system and ensuring patient safety at the same time requires adopting a management system based on well-defined processes. Indeed, the state of São Paulo must comply with Resolution SS-83 of 2015, which can punish a healthcare establishment by means of a financial discount on the value of the medication dispensed through legal means.

In this regard, some important actions were adopted, including the institutionalization of the Health Technology Evaluation Center in 2014, aiming to promote and expand health technology evaluations at teaching and research hospitals, with the responsibility of evaluating requests for health technologies offered to patients within the scope of the health institution. The Health Technology Evaluation Center performs its tasks in accordance with the Pharmacology Commission of the São Paulo Secretary of Health, as stipulated in Resolution SS-54 of May 2012 [13]. For the purposes of this study, the main route of access to health technologies not available in the universal healthcare system was considered cost-effective.

Analyzing prescriptions for medications not available through the universal healthcare system according to the 2022 National List of Essential Medicines offered at the General and Specialty Outpatient Clinic - Men’s Health, the most prescribed medications for ED were PDE-5 inhibitors and testosterone. Tadalafil 5 mg was the most prescribed, accounting for 655 prescriptions and corresponding to 48% of the total number of prescriptions. This medication can be used both to improve sexual performance and treat some cases of benign prostatic hyperplasia, which is a process denominated “optimization” [25].

Currently, the drug sildenafil 20 mg, which belongs to the class of PDE5 inhibitors, is incorporated into the universal healthcare system for pulmonary arterial hypertension, and there has also been evidence of increased access to sildenafil 25 mg and 50 mg for the treatment of systemic sclerosis. It is important to emphasize that there were no records of submission to the National Commission of Incorporation of Technologies in the Universal Healthcare System by the pharmaceutical industry for medications indicated for ED, nor was there any justification for this [6,26-28].

Thus, patients treated by the universal healthcare system for ED are not ensured by public policies to receive drug treatment appropriate for their clinical condition, demonstrating an inconsistency with the National Policy for Comprehensive Men’s Health Care. This underscores the importance of interaction between the Health Technology Evaluation Center and the Pharmacology Commission of the São Paulo State Secretary of Health, which provides patients with cost-effective access to health technologies not available through the universal healthcare system.

Among the access policies, the Generic Medication Law (nº 9787) was created to favor access to medications at a more affordable cost. However, such medications must also be included on the National List of Essential Medicines so that they can be prescribed by a physician within the scope of the universal healthcare system. This is considered paradoxical; as medicine marketed as generic is not on the National List, it cannot be prescribed by a doctor in the universal healthcare system. However, it is worth noting that if access occurs through the courts, the health institution may be forced to offer the medication at a discount, matching that of its generic counterpart [29].

The importance of the Document Screening Sector for Access to Medicines in monitoring administrative processes rejected by the São Paulo State Secretary of Health is highlighted, as this document is essential for patients to take legal action against the state and municipalities. The number of administrative processes sent by the Document Screening Sector for Access to Medicines and approved by the Pharmacology Commission of the São Paulo State Secretary of Health was significant, totaling 85%. This provided patients access to the proposed treatment through these cost-effective actions without the need to resort to legal action for 4,573 tadalafil tablets, 1,206 sildenafil 50 mg tablets, 216 tadalafil 20 mg tablets, 130 sildenafil 25 mg tablets, and 120 testosterone 250 mg/4 mL ampoules.

In addition to providing access to medications, it was necessary to consider the monitoring and engagement of patients from the perspective of care in all stages of the therapeutic chain. The period of medication supply for treatment offered by the São Paulo State Secretary of Health is around four to six months. Therefore, it is necessary to adopt actions, such as preparing the administrative process in a timely manner, correcting and monitoring documents, following up on requests, managing logistics, receiving and storing technologies, dispensing, scheduling infusions that take place at the High-Cost Medication Application Center, monitoring adherence to treatment, and controlling the scheduling of the next appointment. This demonstrates the importance of pharmaceutical care to requesting and monitoring exams in harmony with the frequency of renewals established by the Pharmacology Commission of the São Paulo State Secretary of Health, favoring patient access to the medication.

It is important to emphasize the occurrence of failures in this process, among which we can mention the interruption of treatment due to a shortage of medications, for which responsibility is attributed to the São Paulo State Secretary of Health. Aiming at patient-centered care to avoid the interruption of treatment and ensure access through the courts, it was possible, under some conditions, for the institution to provide the medication.

From the perspective of the present study, the judicialization of medications not available in the universal healthcare system is associated with the unavailability of medications indicated for the treatment of sexual dysfunction, especially ED, in pharmaceutical assistance programs, which makes the work of prescribers in specialized services more difficult. Nonetheless, the present study found that it was possible to provide patients with treatment in a cost-effective manner, with little need to resort to legal action to ensure access.

According to data collected on the judicialization of prescriptions offered at the Regional Medical School Foundation, the largest number of items subject to legal action occurred due to the claim for medications (1,557). However, the work carried out at the outpatient clinic with prescriptions containing medications for ED was relevant, as only a small amount of the dispensations of medications subject to legal action were related to the treatment of ED (0.8%), corresponding to three patients, according to data provided by the judicialization spreadsheet received from the São Paulo State Secretary of Health for the period from 2016 to 2020.

The rate of judicialization was considered low (0.8%) when considering the total number of prescriptions containing specific medications for ED offered to patients (1,377) and the percentage of other medications judicialized in the health institution (64%), as shown in Figure 2.

This work has limitations, making it necessary to expand research on access to medications not yet incorporated into the universal healthcare system in specialized care.

As medications constitute the most widely employed therapeutic intervention and constitute a technology that has a high impact on health spending, it is essential to the sustainability of the universal healthcare system for the process of technological incorporation to be based on criteria that enable the population access to safer, more effective, cost-effective medications to address their main health problems.

## Conclusions

Although medical prescriptions for users diagnosed with ED in outpatient care included drugs not covered by the SUS (Sistema Único de Saúde - Unified Health System), it was observed that the therapeutic choice considered cost-effectiveness criteria and resulted in a low rate of judicialization.

Despite the antagonisms inherent in prescribing medications within the SUS, in accordance with current legislation, collaborative work among team members proved to be fundamental to achieving satisfactory clinical outcomes. The synergistic action enabled the promotion of rational access to medications, reinforcing the importance of interdisciplinarity in patient care and contributing significantly to the sustainability of the public health system. These results indicate that, even in challenging contexts, integrated practices can overcome institutional barriers, amplifying the positive impact on health policies.
